# Systemic immune–inflammation index for predicting postoperative atrial fibrillation following cardiac surgery: a meta-analysis

**DOI:** 10.3389/fcvm.2024.1290610

**Published:** 2024-02-05

**Authors:** Yu-Chou Chen, Chien-Cheng Liu, Hui-Chen Hsu, Kuo-Chuan Hung, Ying-Jen Chang, Chun-Ning Ho, Chung-Hsi Hsing, Ching-Yi Yiu

**Affiliations:** ^1^ Department of Anesthesiology, Chi Mei Medical Center, Tainan, Taiwan; ^2^ Department of Anesthesiology, E-Da Hospital, I-Shou University, Kaohsiung, Taiwan; ^3^ Department of Nursing, College of Medicine, I-Shou University, Kaohsiung, Taiwan; ^4^ School of Medicine, I-Shou University, Kaohsiung, Taiwan; ^5^ Department of Otolaryngology, Kuang Tien General Hospital, Taichung, Taiwan; ^6^ Department of Recreation and Health-Care Management, College of Recreation and Health Management, Chia Nan University of Pharmacy and Science, Tainan, Taiwan; ^7^ Department of Medical Research, Chi-Mei Medical Center, Tainan, Taiwan; ^8^ Department of Otolaryngology, Chi Mei Medical Center, Liouying, Tainan, Taiwan; ^9^ Department of Dental Laboratory Technology, Min-Hwei Junior College of Health Care Management, Liouying, Tainan, Taiwan

**Keywords:** postoperative atrial fibrillation, systemic immune-inflammation index, cardiac surgery, inflammation, cardiopulmonary bypass

## Abstract

**Background:**

Postoperative atrial fibrillation (POAF) is a frequent complication that may increase morbidity and mortality risk following cardiac surgery. The systemic immune–inflammation index (SII) is an emerging biomarker that provides an integrated measure of inflammation by incorporating neutrophil, lymphocyte, and platelet counts. Recent studies have reported associations between elevated SII and increased POAF risk; however, significant heterogeneity exists regarding its predictive efficacy. This meta-analysis aimed to assess SII's diagnostic efficacy for predicting POAF risk.

**Methods:**

To synthesize existing evidence on the ability of perioperative SII for predicting POAF in patients undergoing cardiac surgery, a systematic review and meta-analysis was conducted. In August 2023, a comprehensive literature search was performed to identify relevant studies reporting SII cutoff values with corresponding sensitivity and specificity. The primary aim was to evaluate SII's diagnostic utility for predicting POAF, whereas secondary outcomes included the pooled incidence of POAF and the relationship between the SII and POAF.

**Results:**

Eight studies published between 2021 and 2023 with 3,245 patients were included. Six studies involved coronary artery bypass grafting (CABG) surgery; one encompassed various cardiac procedures, and another focused solely on mitral valve surgery. The pooled incidence of POAF was 23.6% [95% confidence interval (CI), 18.7%–29.2%]. Elevated SII significantly increased the odds of POAF by 3.24-fold (odds ratio, 3.24; 95% CI, 1.6–6.55; *p *= 0.001). SII's pooled sensitivity and specificity for predicting POAF were 0.80 (95% CI, 0.68–0.89) and 0.53 (95% CI, 0.23–0.8), respectively. The SII had moderate predictive accuracy based on a hierarchical summary receiver operating characteristic (HSROC) area under the curve of 0.78 (95% CI, 0.74–0.81). Subgroup analyses, whether focusing on CABG alone or CABG with cardiopulmonary bypass (CPB), both indicated an area under the HSROC curve of 0.78 (95% CI, 0.74–0.81).

**Conclusion:**

Elevated SII is significantly correlated with an increased POAF risk following cardiac surgery, highlighting its utility as a predictive biomarker. Considering its moderate diagnostic accuracy, further research is essential for clarifying SII's clinical effectiveness, either as an independent predictor or combined with other risk factors, for stratifying patients at high POAF risk.

**Systematic Review Registration:**

https://www.crd.york.ac.uk/prospero/, identifier [CRD42023456128].

## 1 Introduction

Postoperative atrial fibrillation (POAF) is the most frequently observed heart rhythm disorder following cardiac surgery (1). Studies have shown that its occurrence rates widely vary from 10% to 65% (2–4). This type of arrhythmia can develop following different types of cardiac surgeries, including coronary artery bypass grafting (CABG), valve repair or replacement, and congenital heart defect repair (5). POAF development has been associated with several adverse consequences, including increased risks of hemodynamic instability, stroke, heart failure, infections, thromboembolic events, renal failure, reduced quality of life, extended hospital stays, and short- and long-term mortality (2, 6–9). Identifying patients at the highest risk for developing POAF could allow for more targeted prophylactic therapy and management to improve outcomes. Despite this, owing to the complex interplay between patient factors, procedural characteristics, and the systemic inflammatory response induced by surgery, POAF prediction remains challenging (5, 10, 11).

In recent years, increasing attention has been paid to hematologic biomarkers that can reflect underlying inflammatory states and may hold prognostic value for various postoperative complications (12–14). The systemic immune–inflammation index (SII), calculated as the product of peripheral neutrophil, platelet, and lymphocyte counts, provides an integrated measure of the inflammatory and prothrombotic response (15–18). Higher SII levels indicate greater systemic inflammation and immune activation. Emerging studies have reported associations between elevated preoperative SII levels and increased POAF risk following cardiac surgery (14, 19–22). Proposed mechanisms include the SII representing increased atrial inflammatory infiltrates and fibrosis, which provide the substrate for new-onset POAF, as well as heightened prothrombotic states that precipitate microthromboses and atrial ischemia, thereby contributing to POAF occurrence (17, 18) (23–25). However, significant between-study heterogeneity exists regarding the predictive efficacy of the SII (14, 19–22). Therefore, to clarify the utility of the SII as a POAF risk stratification tool among patients undergoing cardiac surgery, additional investigations are needed.

Cardiac procedures including CABG can induce a strong systemic inflammatory response, provoked by factors such as surgical trauma, use of cardiopulmonary bypass (CPB), ischemia–reperfusion damage, and hemodilution (26, 27). Considering the usefulness of the SII as an inflammation biomarker, we conducted a systematic review and meta-analysis synthesizing existing evidence on the ability of perioperative SII, which is widely available and inexpensive, for predicting POAF following cardiac surgery. By pooling data across studies, we aimed to provide enhanced precision in estimating the predictive value of the SII. Our findings will help determine whether the SII could serve as a useful prognostic biomarker for identifying patients at increased POAF risk following cardiac procedures who may benefit from targeted preventive therapies.

## 2 Methods

### 2.1 Data source and protocol registration

This review adhered to the PRISMA guidelines and was duly registered in PROSPERO (registration number: CRD42023456128). To identify relevant studies, a comprehensive literature search was performed in MEDLINE, Embase, Google Scholar, and the Cochrane Library from inception to August 2023. The specific search strategies included a combination of controlled vocabulary terms (MeSH and Emtree) and keywords related to the index test (e.g., “systemic immune–inflammation index” and “SII”) and the target condition (e.g., “atrial fibrillation”). No geographic or language restrictions were imposed. Additional eligible studies were identified by hand-searching reference lists of relevant articles. For one of the databases (i.e., MEDLINE), Table 1 summarizes the details of search strategies.

**Table 1:** Search strategy for medline.

1	(“coronary artery bypass surger*” or “cardiopulmonary bypass surger*” or “cardiovascular surger*” or “cardiac surger* “ or “CABG” or “off-pump coronary artery surger*” or “coronary artery bypass graft surger*” or “Heart Surger*” or “Cardiac Surgical Procedure* “ or “(Aortic or Mitral or Heart Valve Prosthesis Implantation or Aortic Valve or Mitral Valve) adj4 (procedure* or operation* or surger*)”).mp.
2	exp “Cardiac Surgical Procedures"/
3	(Systemic Immune-Inflammation Index).mp.
4	(“Atrial Fibrillation” or “Af”).mp.
5	exp “Atrial Fibrillation"/
6	(1 or 2) and 3 and (4 or 5)

### 2.2 Study selection based on inclusion and exclusion criteria

Two independent authors reviewed the titles and abstracts of the retrieved records to determine potential eligibility. Following duplicate removal, the full texts of articles were evaluated on the basis of the predefined inclusion and exclusion criteria. Studies were considered eligible if they (1) enrolled adult patients undergoing cardiac surgery with or without the use of CPB; (2) assessed either preoperative or postoperative SII as a diagnostic predictor of POAF; (3) reported the cutoff values for SII along with the associated risk estimates or diagnostic profiles, such as sensitivity and specificity; and (4) utilized a cohort, case–control, randomized-controlled, or cross-sectional design. Reviews, editorials, case reports, conference abstracts, and pediatric studies were excluded. A further exclusion was made for studies that focused on patients who did not undergo cardiac surgery. Any disagreements regarding study inclusion were addressed through consensus discussion with a third researcher.

### 2.3 Data extraction

Two independent reviewers used a standardized form for extracting relevant data from the included studies. The following were the extracted details: author's information, country, number of participants, patient demographics (e.g., gender and age), sensitivity, specificity, SII cutoff values, POAF incidence, and surgery type. Any discrepancies in data extraction were resolved through collaboration and consensus between the two authors. If needed, study authors were contacted for clarification or to obtain any missing information.

### 2.4 Primary and secondary outcomes

The main focus was on assessing the diagnostic effectiveness of the SII for predicting POAF following cardiac surgery, constituting the primary outcome. Secondary outcomes consisted of the pooled incidence of POAF and the relationship between the SII and POAF.

### 2.5 Quality assessment

To evaluate the methodological quality and bias risk of the included studies by two independent reviewers, the QUADAS-2 was used. This tool consists of the following four key domains: patient selection, index test, reference standard, and flow and timing. Each domain was assessed in terms of risk of bias, and the first three domains were also rated for concerns regarding applicability. Any conflicts in the quality assessment were resolved by consensus. The overall study quality was summarized graphically.

### 2.6 Statistical analysis

Using the random-effects model, we computed the pooled sensitivity, specificity, odds ratios (ORs), positive likelihood ratios (PLRs), negative likelihood ratios (NLRs), and mean differences (MDs), along with their corresponding 95% confidence intervals (CIs). We computed the area under the hierarchical summary receiver operating characteristic (HSROC) curve to assess the overall diagnostic test accuracy. Moreover, the I^2^ statistic was used to explore between-study heterogeneity, considering values >75% as indicative of significant heterogeneity. Potential publication bias was evaluated through visual scrutiny of Deeks' funnel plot. Subgroup analyses were conducted considering two criteria: the type of surgical procedure, with a specific focus on cases in which only CABG was performed, and instances involving the use of CPB. In the first subgroup analysis, our focus was exclusively on data from studies centered on CABG surgery. For the second subgroup analysis, we focused on data derived from studies that specifically involved CABG procedures that incorporated the use of CPB. The certainty of evidence for the predictive efficacy of SII for POAF was evaluated using the Grading of Recommendations, Assessment, Development and Evaluation (GRADE) methodology (28). The certainty assessment examines five domains: risk of bias, indirectness, inconsistency, imprecision, and publication bias. Each domain was judged as not serious, serious, or very serious. The certainty rating starts at high for comparative test accuracy studies but may be downgraded by one or two levels per domain if serious or very serious concerns are identified. The overall certainty is then determined after considering ratings across all domains. All statistical analyses were performed using Stata version 16.0 and Review Manager (RevMan) 5.4 (The Cochrane Collaboration, 2020). A significance level of *p* < 0.05 was established for all statistical analyses to determine statistical significance.

## 3 Results

### 3.1 Selection and characteristics of studies

**Figure 1:**
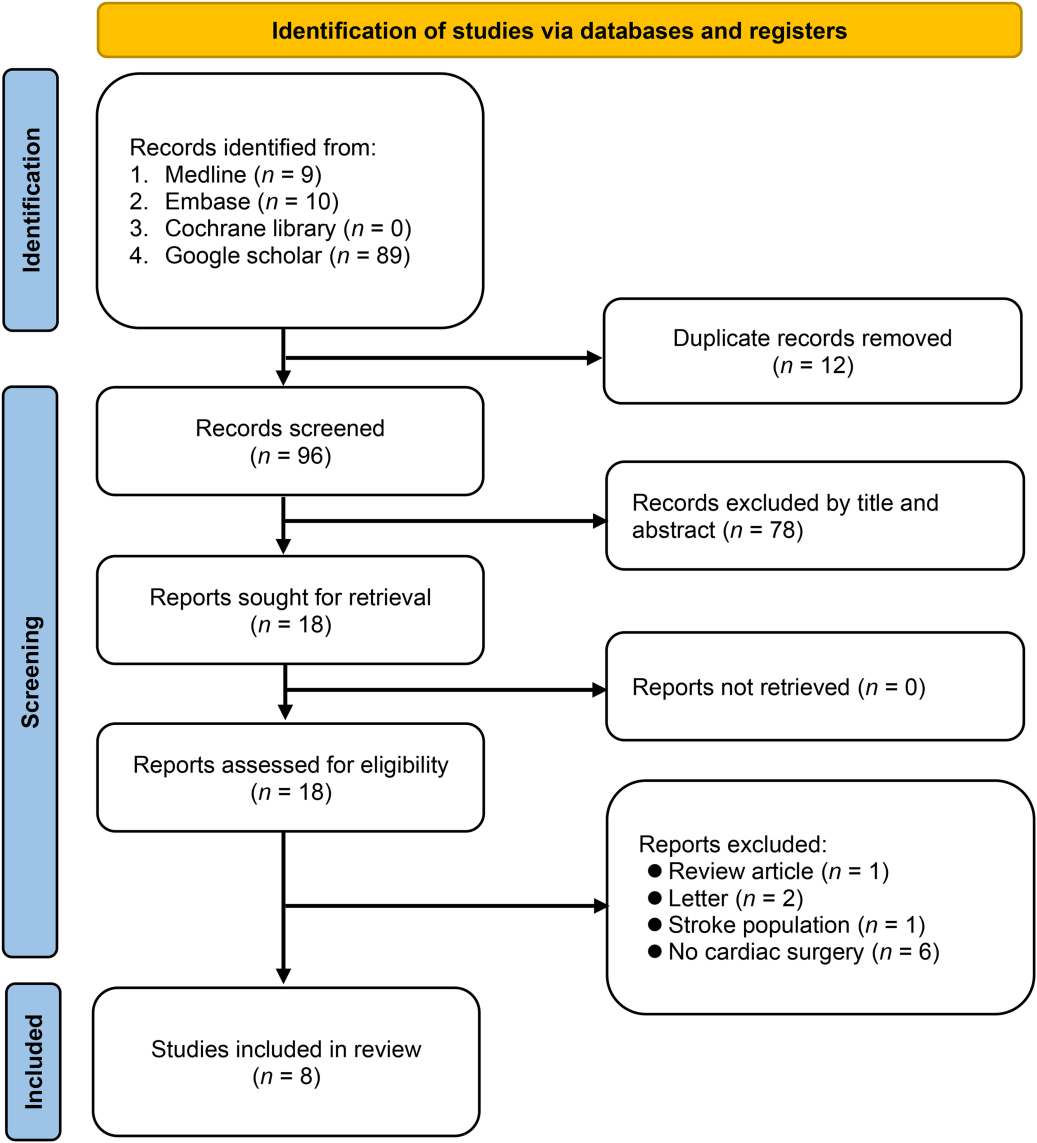
Flowchart of study selection.

The literature search spanned four databases—MEDLINE, Embase, Cochrane Library, and Google Scholar—and yielded 108 records (Figure 1). After removing 12 duplicates, 96 records were screened on the basis of predefined inclusion criteria. This process excluded 78 records, leaving 18 for full-text review. Of these, eight were excluded for reasons such as being review article (*n* = 1), letters (*n* = 2), stroke population (*n* = 1), or irrelevant to cardiac surgery (*n* = 6). Ultimately, eight studies involving 3,245 patients were included in this meta-analysis (Figure 1) (14, 19–22, 29–31).

The main characteristics of the included studies are summarized in Table 2. The sample size ranged from 116 to 1,007 patients, with a mean or median age across studies ranging from 59 to 70 years. One study (14) did not explicitly report the sensitivity and specificity of SII for predicting POAF. Instead, it provided the risk estimate of POAF in patients with high SII values (14). The majority of study participants were male, comprising 35.2%–78.4% of patients. Six studies enrolled patients undergoing CABG (14, 20, 21, 29–31), whereas one study also included those undergoing other cardiac surgical procedures, including valve replacements (22). Another study only enrolled patients undergoing mitral valve surgery (19). Seven studies in our meta-analysis focused on preoperative measurements of the SII. In contrast, one study (19) was unique in its focus on data collected on the seventh postoperative day. POAF incidence varied from 18% to 43% in the included studies. Cutoff values used for the SII as a POAF predictor differed across studies (range, 545–1,696). The median cutoff value was 836.9, calculated as the average of 807.8 and 866.04.

**Table 2:** Characteristics of studies (*n* = 8).

First Author (Year)	Age (years)	Study Number	Male (%)	Surgery	CPB	AF Incidence	AF+	AF-	Sensitivity	Specificity	SII Cut-off	Country
Ata (2021)	59 (52.5–62)	283	65.1%	CABG	Yes	25.4%	72	211	72.2%	74.4%	986^b^	Turkey
Dey (2021)^d^	67 ± 8	1,007	72.3%	CABG	No	20.4%	158	849	NA	NA	878.06^b^	India
Hinoue (2023)	70 (60–75)	212	67%	CABG^a^	Yes	43%	90	122	71%	81%	545^b^	Japan
Luo (2022)	59.6 ± 6.4	122	35.2%	MVS	Yes	18%	22	100	63.6%	68%	1,696^c^	China
Selcuk (2021)	61 ± 10	391	76.5%	CABG	Yes	26%	97	294	60.8%	80.9%	807.8^b^	Turkey
Topal (2022)	62 (35–87)	722	63.8%	CABG	No	23.8%	172	550	86.6%	29.3%	706.7^b^	Turkey
Uğuz (2022)	61 ± 10	116	78.4%	CABG	Yes	22%	26	90	92.31%	1.11%	866.04^b^	Turkey
Yilmaz (2021)	59 (52.5–62)	392	71.2%	CABG	Yes	20.4%	80	312	85%	61.2%	712.8^b^	Turkey

CABG, coronary artery bypass grafting; MVS, mitral valve surgery; AF, atrial fibrillation; SII, Systemic Immune-Inflammation Index; CPB, cardiopulmonary bypass.

^a^ CABG with or without valve surgery.

^b^ Preoperative.

^c^ postoperative day 7.

^d^ This study reported that participants with an SII exceeding the established cutoff experienced a notably higher incidence of developing AF compared to those below this threshold.

**Figure 2:**
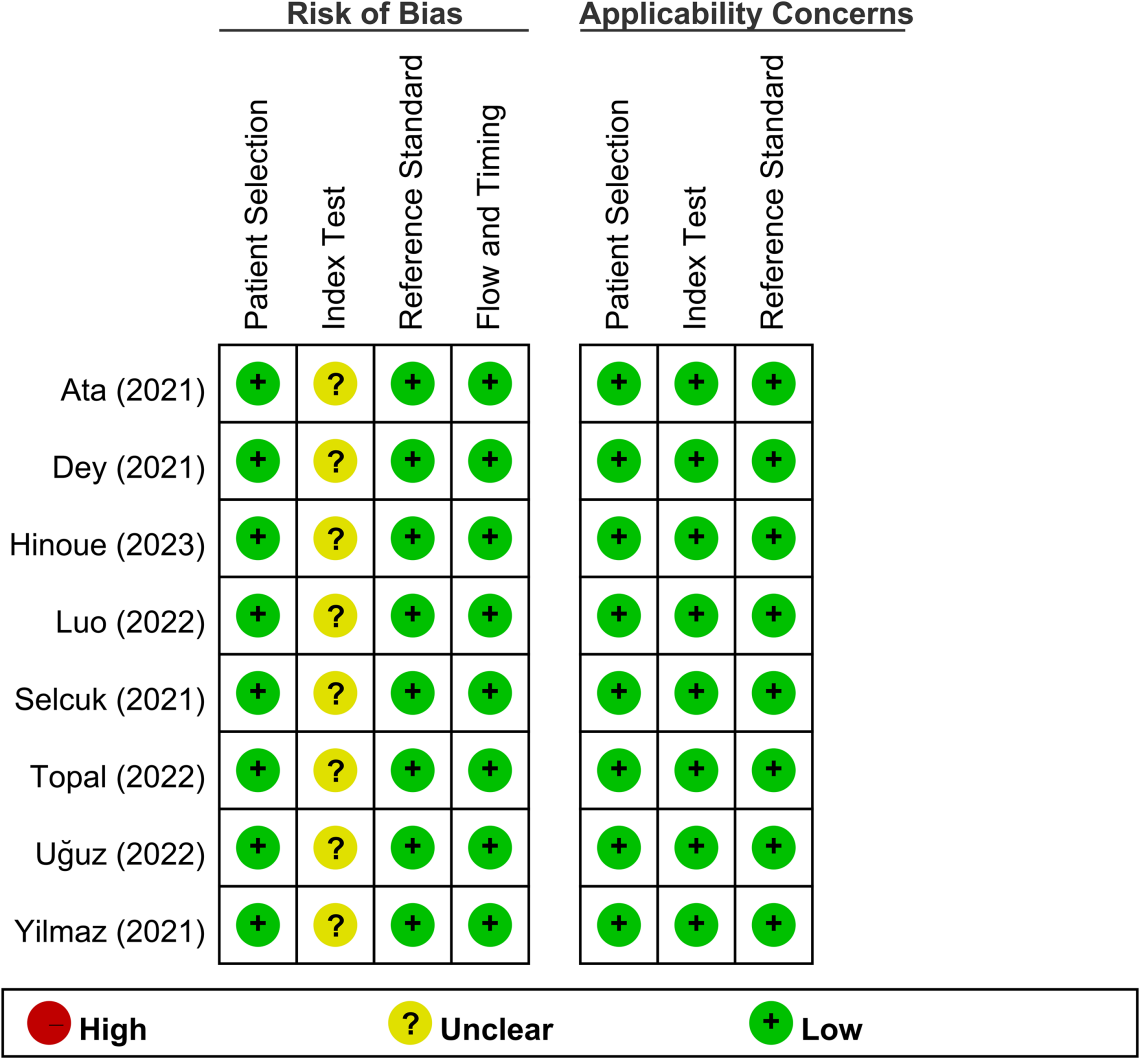
Assessment of risk of bias for the included studies.

The risk of bias and concerns regarding applicability across all examined studies are delineated in Figure 2. Regarding bias risk, the criteria of patient selection, reference standard, flow, and timing were uniformly judged to have a low risk across all included studies. Nevertheless, uncertainty existed in the index test domain for eight studies, which was primarily attributed to the absence of predefined cutoff values for the SII. Regarding concerns related to applicability, the collective body of studies was assessed to have a low risk of bias, thereby bolstering the generalizability of the findings.

### 3.2 POAF incidence and the relationship between the SII and POAF

**Figure 3:**
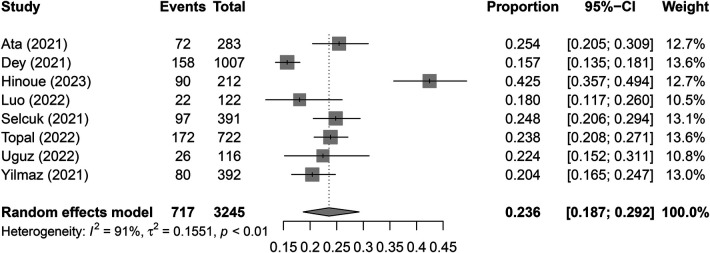
Pooled incidence of postoperative atrial fibrillation (POAF) following cardiac surgery. Incidence: 23.6% (95% CI: 18.7%–29.2%). CI, confidence interval.

**Figure 4:**
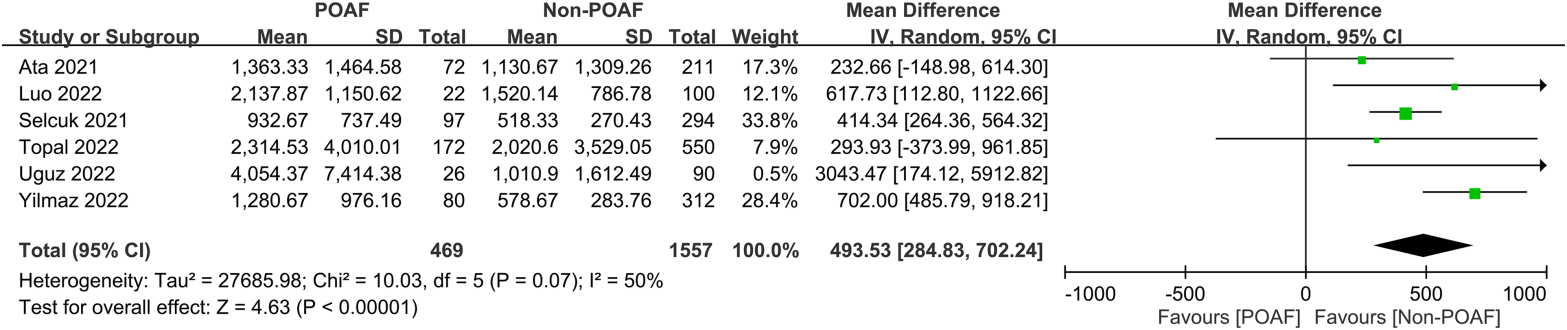
Forest plot comparing systemic immune–inflammation index (SII) values between patients with and without postoperative atrial fibrillation (POAF) following cardiac surgery.

**Figure 5:**
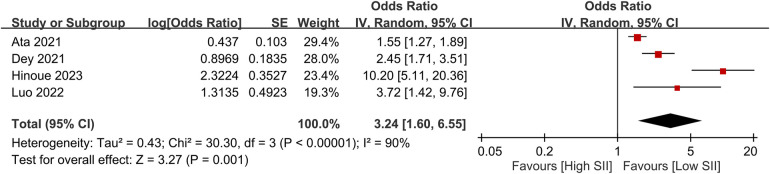
Forest plot showing the odds ratio for postoperative atrial fibrillation (POAF) in patients with high versus low SII levels.

The pooled incidence of POAF was 23.6% (95% CI, 18.7%–29.2%; *I*^2^ = 91%) across the included studies (Figure 3) (14, 19–22, 29–31). Patients who developed POAF had significantly higher SII values than those who did not, with a pooled MD of 493.53 (95% CI, 284.83–702.24; *p *< 0.00001) (Figure 4) (19–21, 29–31). The odds of developing POAF were 3.24-fold higher in patients with high SII levels (OR, 3.24; 95% CI, 1.6–6.55; *p *= 0.001) (Figure 5) (14, 19, 20, 22).

### 3.3 Diagnostic efficacy of the SII for predicting POAF

**Figure 6:**
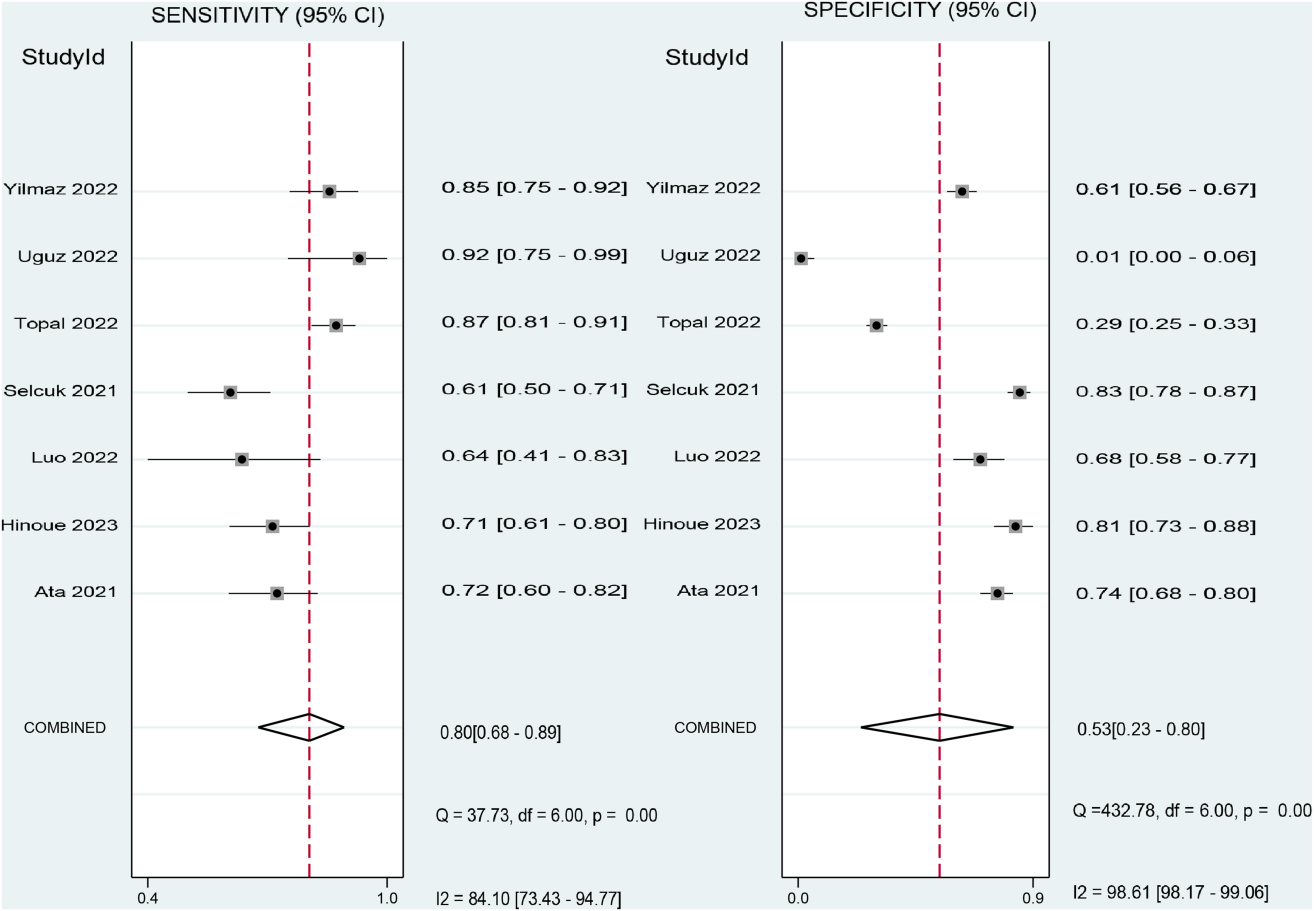
Forest plot showing the pooled sensitivity and specificity of the systemic immune–inflammation index (SII) for predicting postoperative atrial fibrillation (POAF) following cardiac surgery.

**Figure 7:**
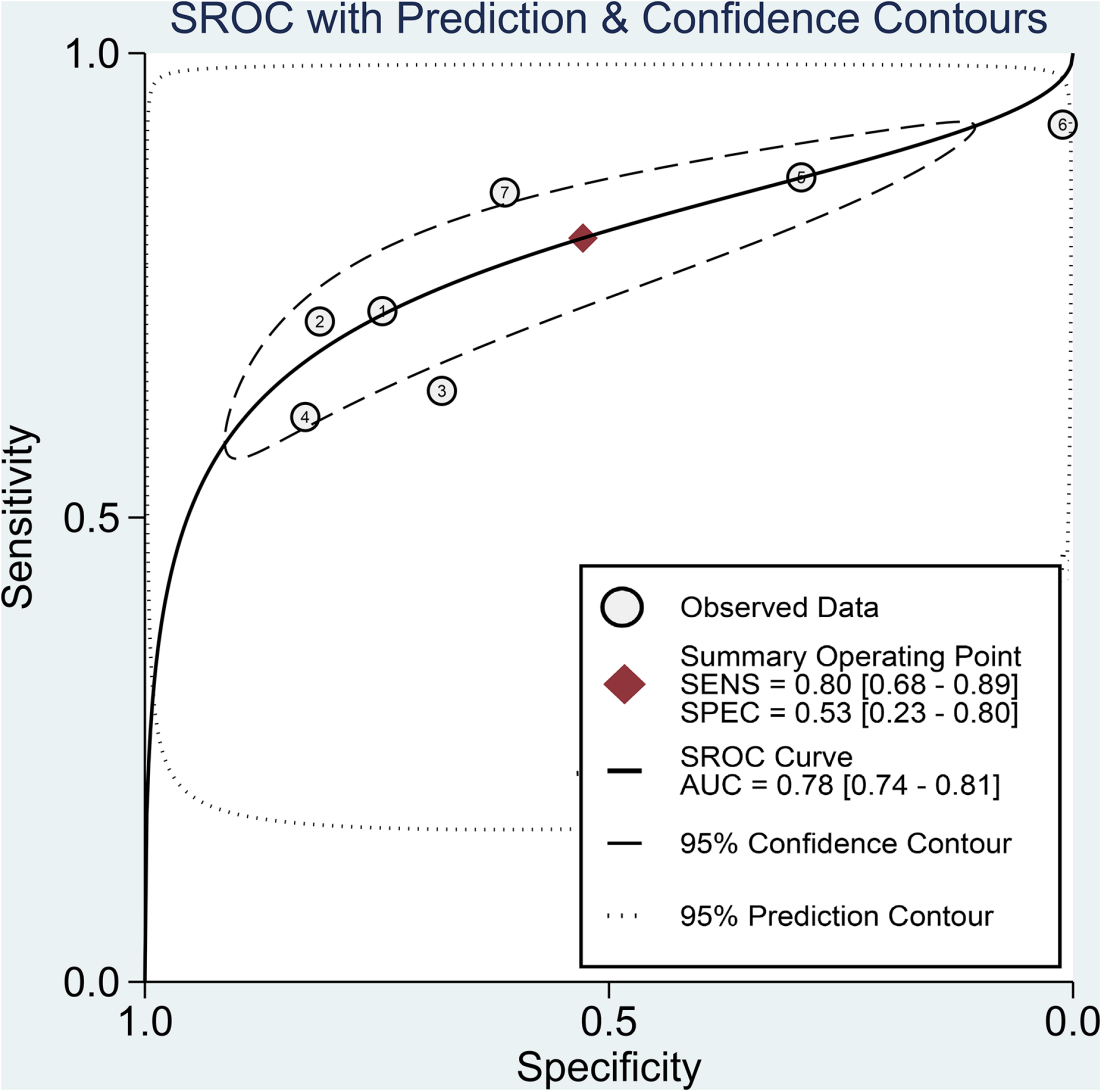
Hierarchical summary receiver operating characteristic (HSROC) curve demonstrating the predictive efficacy of the systemic immune–inflammation index (SII) for predicting postoperative atrial fibrillation (POAF) following cardiac surgery. The HSROC curve plots the pooled sensitivity and specificity estimates, with the black circle representing the summary operating point. The curve itself summarizes the overall diagnostic accuracy, whereas the area under the HSROC curve quantifies the diagnostic test performance. The closer the curve is to the upper left corner, the higher the overall accuracy of the test. The size of the black circles reflects the statistical weight of each study in the meta-analysis. The dashed lines represent the confidence region around the summary operating point. This HSROC curve has an area under the curve of 0.78, suggesting moderate accuracy of the SII for predicting new-onset AF following cardiac surgery.

**Figure 8:**
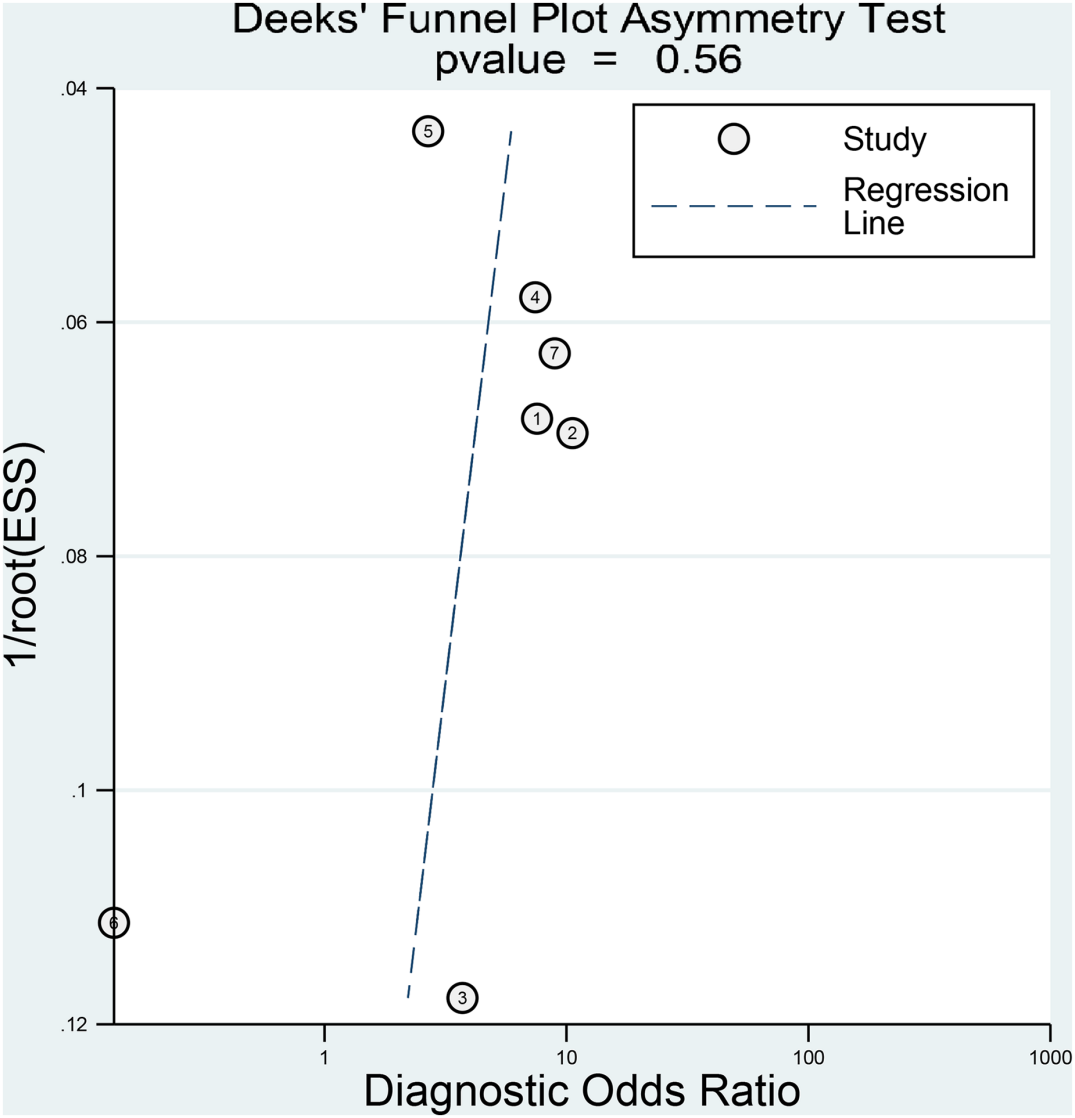
Deeks’ funnel plot assessing publication bias in studies reporting the predictive value of the systemic immune–inflammation index (SII) for postoperative atrial fibrillation (POAF) following cardiac surgery.

The pooled sensitivity and specificity of the SII for predicting POAF were 0.80 (95% CI, 0.68–0.89; *I*^2^ = 84.1%) and 0.53 (95% CI, 0.23–0.8; *I*^2^ = 98.61%), respectively (Figure 6) (19–22, 29–31). Sensitivity varied from 60.8% to 92.31%, and specificity ranged from 1.1% to 81% among individual studies. Significant between-study heterogeneity was noted. The area under the HSROC curve was 0.78 (95% CI, 0.74–0.81) (Figure 7), suggesting that the SII has moderate overall diagnostic accuracy in distinguishing between patients who will and will not develop POAF following cardiac surgery. Deeks' funnel plot test indicated that the chance of publication bias is low, as shown by a *p*-value of 0.26 (Figure 8).

### 3.4 Fagan nomogram for post-test probabilities

**Figure 9:**
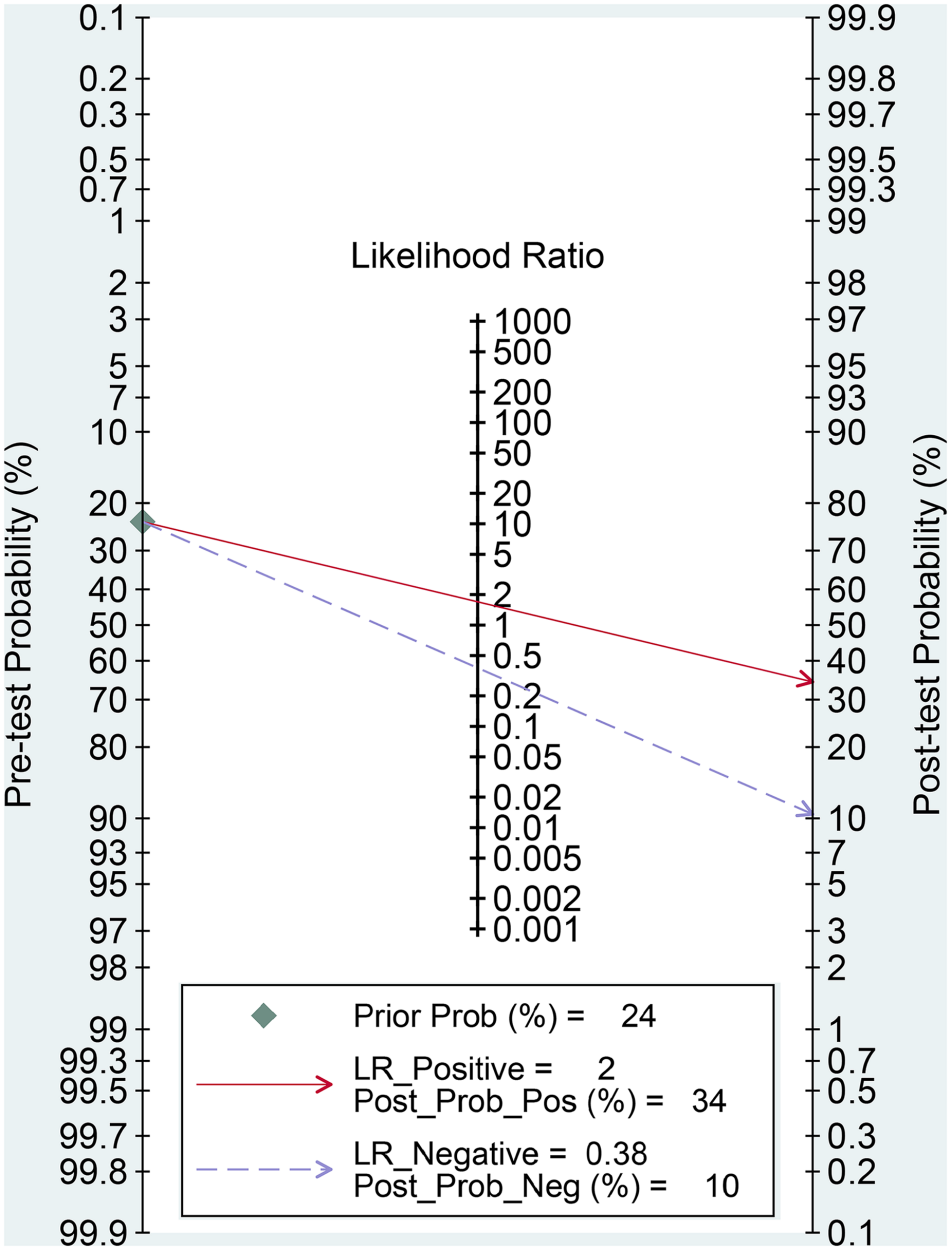
Fagan nomogram for assessing the clinical utility of the systemic immune–inflammation index (SII) for predicting postoperative atrial fibrillation (POAF) following cardiac surgery.

The effectiveness of the SII for predicting POAF was assessed through Fagan nomograms. The test yielded a PLR and NLR of 2 and 0.38, respectively. Provided an initial likelihood of 24% for POAF occurrence, using the SII diagnostic test modified this to a 34% probability with a positive test outcome and a 10% probability with a negative test outcome (Figure 9).

### 3.5 Subgroup analysis

**Figure 10:**
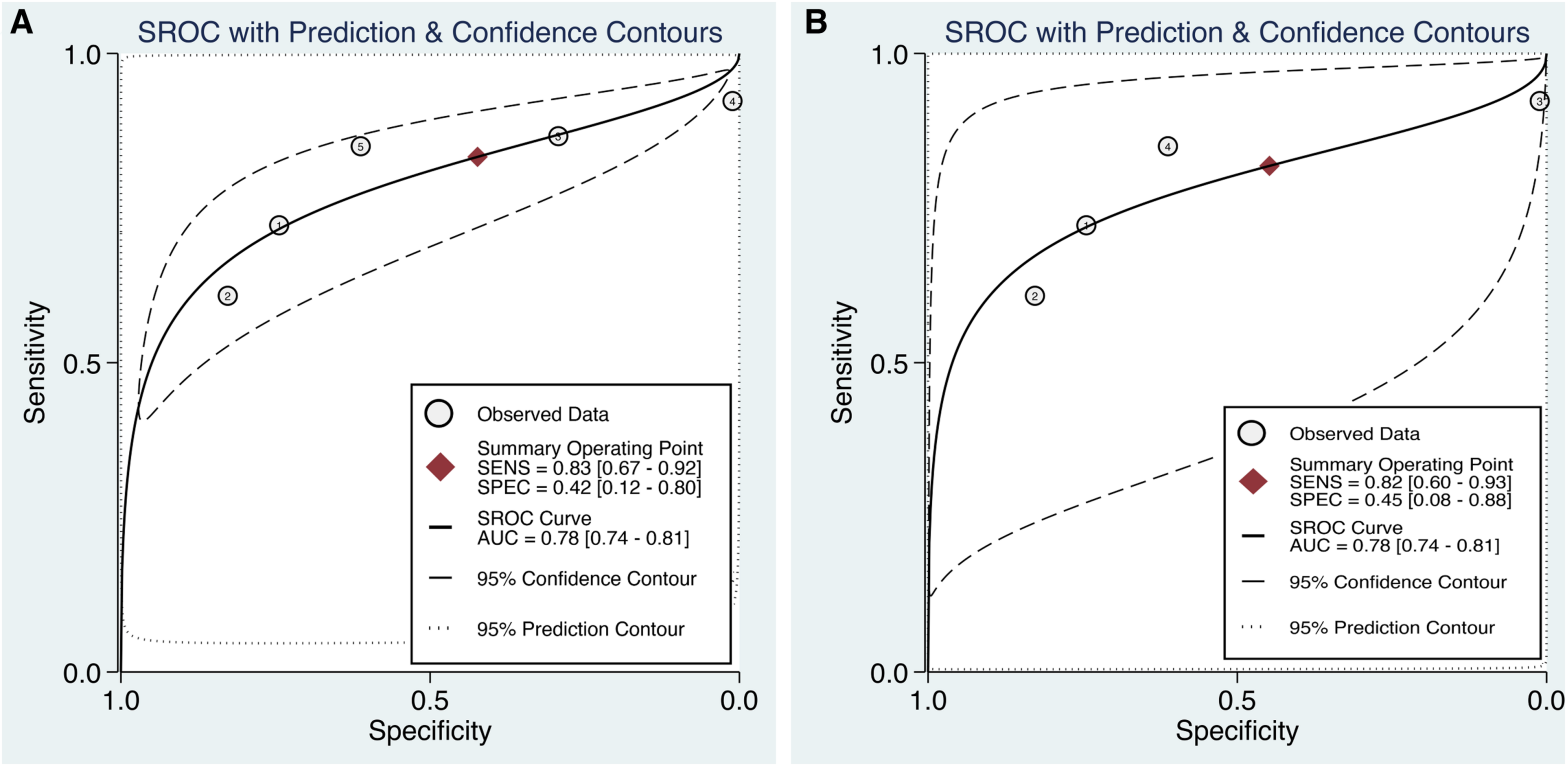
Subgroup analyses in the predictive efficacy of the systemic immune–inflammation index (SII) for predicting postoperative atrial fibrillation (POAF) based on (a) the type of surgical procedure, with a specific focus on cases in which only coronary artery bypass grafting (CABG) surgery was performed, and (b) CABG surgery involving the use of cardiopulmonary bypass (CPB). Hierarchical summary receiver operating characteristic (HSROC) curve demonstrating The HSROC curve plots the pooled sensitivity and specificity estimates, with the black circle representing the summary operating point. The curve itself summarizes the overall diagnostic accuracy, whereas the area under the HSROC curve quantifies the diagnostic test performance. The closer the curve is to the upper left corner, the higher the overall accuracy of the test. The size of the black circles reflects the statistical weight of each study in the meta-analysis. The dashed lines represent the confidence region around the summary operating point.

Subgroup analysis based on the type of surgical procedure, with a specific focus on cases in which only CABG was performed, is show in Figure 10A. These studies exclusively used preoperative SII values as a predictive tool for POAF (20, 21, 29–31). The pooled sensitivity and specificity of the SII for predicting POAF were 0.83 (95% CI, 0.67–0.92; *I*^2^ = 88.13%) and 0.42 (95% CI, 0.12–0.8; *I*^2^ = 98.96%), respectively. Furthermore, the area under the HSROC curve was 0.78 (95% CI, 0.74–0.81) (Figure 10A).

Subgroup analysis focused on CABG with the use of CPB revealed that the pooled sensitivity and specificity of the SII for predicting POAF were 0.82 (95% CI, 0.60–0.93; *I*^2^ = 85.54%) and 0.45 (95% CI, 0.08–0.88; *I*^2^ = 98.66%), respectively. The area under the HSROC curve was 0.78 (95% CI, 0.74–0.81) (Figure 10B).

### 3.6 Certainty of evidence

The certainty of evidence for the predictive efficacy of SII was judged to be low. The evidence was judged to have no serious risk of bias and no serious indirectness. However, serious concerns were identified regarding inconsistency, with significant heterogeneity found in sensitivity and specificity estimates (*I*^2^ > 75%), and imprecision due to wide confidence intervals across accuracy measures. No significant publication bias was detected. Overall, the evidence was rated down by one level each for inconsistency and imprecision, resulting in low certainty evidence.

## 4 Discussion

In this meta-analysis involving eight studies and 3,245 patients who underwent cardiac procedures, the pooled incidence of POAF was 23.6%. Patients who developed POAF had significantly higher perioperative SII values than those who did not develop AF (MD, 493.53). The odds of developing POAF were 3.24-fold higher in patients with elevated SII. Diagnostic accuracy measures showed that the pooled sensitivity and specificity of the SII for predicting POAF were 0.80 and 0.53, respectively, with an area under the HSROC curve of 0.78, indicating moderate diagnostic accuracy. However, significant between-study heterogeneity was observed, necessitating caution in the interpretation and generalization of these findings.

POAF is a frequently encountered complication following cardiac procedures, with incidence rates ranging from 20% to 40% (32, 33). In a meta-analysis involving 155,575 patients, POAF incidence occurred in 36,988 patients, with an incidence rate of 23.7% (34). POAF incidence in the current meta-analysis was 23.5%, which is consistent with that reported in the current literature. The majority of POAF cases spontaneously reverted to sinus rhythm before hospital discharge (34). In a single-center study of 7,115 patients undergoing isolated CABG, several etiological factors have been implicated in POAF occurrence, including advancing age, New York Heart Association (NYHA) class III or IV, male gender, smoking history, and prior myocardial ischemia (35). Furthermore, compared with other factors such as male gender and NYHA class III/IV, advancing age was identified as a stronger POAF predictor (35). Not only is POAF linked with extended hospital stays and higher in-hospital mortality but it also exacerbates the risk of long-term mortality (34, 36). Additionally, POAF following cardiac surgery is associated with a fivefold increase in the risk of permanent atrial fibrillation (37).

In the current meta-analysis, diagnostic accuracy measures showed that the pooled sensitivity and specificity of the SII for predicting POAF were 0.80 and 0.53, respectively, with an area under the HSROC curve of 0.78, indicating moderate diagnostic accuracy. The diagnostic efficacy of the SII for predicting POAF can likely be attributed to the index's incorporation of key inflammatory markers, including neutrophils, lymphocytes, and platelets. These components provide an integrated measure of systemic inflammation, which is a known contributor to POAF initiation and progression, particularly in the post-cardiac surgery setting (38, 39). Provided this mechanistic rationale, further studies should aim to elucidate the exact inflammatory pathways involved in POAF development and how the SII may interact with these processes. This could potentially lead to targeted anti-inflammatory therapeutic strategies for reducing POAF incidence in patients undergoing cardiac surgery.

Several risk prediction models for POAF have been developed on the basis of epidemiologic studies rather than pathophysiologic mechanisms (e.g., inflammation). For example, Mahoney et al. conducted a study investigating POAF predictors in a large cohort of over 10,000 patients undergoing cardiac surgery (40). They developed three distinct predictive models, each with areas under the receiver operating characteristic curve (AUC-ROC) of 0.67, 0.65, and 0.64, respectively (40). In a study of 7,115 isolated patients with CABG, Thorén et al. attempted to identify individuals at high risk for developing POAF and observed their final predictive model to have moderate efficacy with an AUC-ROC of 0.62 (35). A risk score for POAF (POAF score) was derived by incorporating demographics, comorbid conditions, and operative data from 17,262 adult patients undergoing cardiac surgery; however, its predictive efficacy was moderate, as evidenced by an AUC-ROC of 0.64 in the validation cohort (41). In light of these findings, employing the SII for predicting POAF on the basis of its physiological mechanisms seems to present clinical advantages over pre-existing predictive models. The use of the SII in perioperative settings could facilitate more precise informed consent discussions by offering patients a clearer understanding of their risk for POAF. Additionally, such risk stratification could enable the targeted application of prophylactic interventions, including antiarrhythmic medications, for patients identified as being at elevated risk.

The inflammatory response to cardiac surgery varies depending on whether CPB is used (42). The use of CPB can indeed alter the magnitude of the systemic inflammatory response, which may in turn affect postoperative outcomes, including the development of POAF. In light of this, we conducted a subgroup analysis focused solely on studies involving CABG procedures that employed CPB, revealing that the area under the HSROC curve was 0.78. Given that the area under the HSROC curve is consistent in both the overall and subgroup analyses, this finding suggests that the preoperative inflammatory response may indeed serve as a primary determinant in the incidence of POAF.

In the current meta-analysis, patients with the SII were 3.24-fold more likely to develop POAF, indicating the potential of the SII as a robust predictive marker. In a previous meta-analysis involving 24 studies and 36,834 participants, various comorbidities including heart failure, chronic obstructive pulmonary disease, hypertension, and myocardial infarction were identified as POAF predictors (43). While these factors are statistically significant, their ORs range only between 1.18 and 1.56, indicating a relatively weak association with POAF. In a single-center study involving 7,115 consecutive patients who underwent isolated CABG, advancing age was identified as a significant POAF predictor (35). Specifically, when compared with patients aged <50 years, those aged between 51 and 60, 61–70, and 71–80 years had ORs for POAF development of 2, 3.8, and 5.5, respectively (35). These data indicate that age may serve as a strong POAF predictor, even surpassing other comorbidities and markers, including the SII. Consequently, age should be an integral part of comprehensive predictive models for POAF, potentially in combination with other significant predictors, including the SII, to enhance both patient risk stratification and targeted prophylactic interventions.

Other inflammation-related biomarkers that potentially predict POAF included neutrophil–lymphocyte ratio (NLR), platelet-to-lymphocyte ratio (PLR), and C-reactive protein (CRP) (13, 44–45). However, limited evidence suggested that increased preoperative PLR is not independently associated with POAF in patients undergoing isolated CABG (44). Although high CRP levels were associated with greater odds of POAF development, their associations were also weak (e.g., OR of 1.31). In a meta-analysis comprising 12 studies with 9,262 participants, increased NLR was observed to be a significant POAF predictor, with a pooled OR of 1.42 (13). The relatively high OR of 3.24 for the SII in the current meta-analysis compared with 1.42 for NLR suggests a stronger association of the SII with POAF, thereby offering critical insights for enhancing preoperative risk stratification.

While emerging studies have reported associations between elevated preoperative SII levels and increased POAF risk following cardiac surgery (14, 19–22), our systematic review and meta-analysis aimed to provide new insights beyond prior studies in several ways. First, by pooling data across studies, we enhanced the statistical power and precision to quantify the relationship between the SII and POAF risk. Second, we evaluated the predictive performance of the SII by synthesizing accuracy metrics such as sensitivity, specificity, and ROC curves, which has not been done previously. Third, by including recently published studies up to August 2022, we provided an updated synthesis of the latest evidence. Therefore, compared to previous studies that established a link between a higher SII and POAF (14, 19–22), our meta-analysis expands on this by more precisely determining the strength of association, predictive utility, POAF incidence, and related metrics, thereby elucidating SII's clinical value of the SII as a prognostic biomarker for POAF risk stratification.

The overall certainty of evidence for the predictive efficacy of SII was low based on the GRADE methodology. The low certainty indicates that while the current body of evidence suggests SII may be useful for POAF risk stratification, further research is likely to improve our confidence in the effect estimates and predictive utility. In particular, additional comparative studies with larger sample sizes and standardized cutoff values for SII would enhance precision and consistency. Nevertheless, the emerging evidence indicates SII warrants further investigation as a prognostic biomarker that provides an integrated measure of the inflammatory state among cardiac surgery patients.

The current meta-analysis had some limitations that warrant careful consideration. First, the inclusion of only eight studies with a total of 3,245 patients may limit the statistical power and generalizability of the findings. Multicenter trials with larger sample sizes would strengthen the reliability and generalizability of these results. Second, as advancing age and gender are potential POAF predictors, the considerable variability in age (e.g., 59–70 years) and male gender (e.g., 35.2%–78.4%) across included studies may be a source of bias. Third, the types of cardiac surgeries undertaken by patients were not uniform across the included studies; six focused on CABG, one included other cardiac procedures, and another was limited to mitral valve surgery. Fourth, no consensus was noted regarding the cutoff values for the SII, with studies employing a range of values between 545 and 1,696. The inconsistency across studies poses challenges for subsequent clinical application. These limitations necessitate cautious interpretation of the findings and underscore the need for larger, more homogeneous studies for more robust conclusions.

## 5 Conclusion

In this meta-analysis of eight studies encompassing 3,245 patients undergoing cardiac surgery, where myocardial revascularization was predominant, we found that the pooled incidence of POAF was 23.6%. Elevated SII increased the odds of POAF by 3.24-fold and had a sensitivity and specificity of 0.80 and 0.53, respectively, with moderate diagnostic accuracy. Owing to significant heterogeneity and the limited number of studies currently available, additional studies to corroborate and elaborate on these initial observations are required.

## Data Availability

The original contributions presented in the study are included in the article/Supplementary Material, further inquiries can be directed to the corresponding author.

## References

[1] Woldendorp K Farag J Khadra S Black D Robinson B Bannon P. Postoperative atrial fibrillation after cardiac surgery: a meta-analysis. Ann Thorac Surg. (2021) 112:2084–93. 10.1016/j.athoracsur.2020.10.055 33340521

[2] Wang MK Meyre PB Heo R Devereaux PJ Birchenough L Whitlock R Short-term and long-term risk of stroke in patients with perioperative atrial fibrillation after cardiac surgery: systematic review and meta-analysis. CJC Open. (2022) 4:85–96. 10.1016/j.cjco.2021.09.011 35072031 PMC8767142

[3] Shen J Lall S Zheng V Buckley P Damiano RJ Jr. Schuessler RB. The persistent problem of new-onset postoperative atrial fibrillation: a single-institution experience over two decades. J Thorac Cardiovasc Surg. (2011) 141:559–70. 10.1016/j.jtcvs.2010.03.011 20434173 PMC2917532

[4] Maisel WH Rawn JD Stevenson WG. Atrial fibrillation after cardiac surgery. Ann Intern Med. (2001) 135:1061–73. 10.7326/0003-4819-135-12-200112180-00010 11747385

[5] McIntyre WF. Post-operative atrial fibrillation after cardiac surgery: challenges throughout the patient journey. Front Cardiovasc Med. (2023) 10:1156626. 10.3389/fcvm.2023.1156626 36960472 PMC10027741

[6] Taha A Nielsen SJ Franzén S Rezk M Ahlsson A Friberg L Stroke risk stratification in patients with postoperative atrial fibrillation after coronary artery bypass grafting. J Am Heart Assoc. (2022) 11:e024703. 10.1161/JAHA.121.024703 35574947 PMC9238552

[7] Saxena A Dinh DT Smith JA Shardey GC Reid CM Newcomb AE. Usefulness of postoperative atrial fibrillation as an independent predictor for worse early and late outcomes after isolated coronary artery bypass grafting (multicenter Australian study of 19,497 patients). Am J Cardiol. (2012) 109:219–25. 10.1016/j.amjcard.2011.08.033 22011556

[8] Alghosoon H Arafat AA Albabtain MA Alsubaie FF Alangari AS. Long-term effects of postoperative atrial fibrillation following mitral valve surgery. J Cardiovasc Dev Dis. (2023) 10(7):302. 10.3390/jcdd10070302 37504558 PMC10380686

[9] Mariscalco G Klersy C Zanobini M Banach M Ferrarese S Borsani P Atrial fibrillation after isolated coronary surgery affects late survival. Circulation. (2008) 118:1612–8. 10.1161/CIRCULATIONAHA.108.777789 18824644

[10] Ishida K Kimura F Imamaki M Ishida A Shimura H Kohno H Relation of inflammatory cytokines to atrial fibrillation after off-pump coronary artery bypass grafting. Eur J Cardiothorac Surg. (2006) 29:501–5. 10.1016/j.ejcts.2005.12.028 16439145

[11] Ihara K Sasano T. Role of inflammation in the pathogenesis of atrial fibrillation. Front Physiol. (2022) 13:862164. 10.3389/fphys.2022.862164 35492601 PMC9047861

[12] Altieri C Pisano C Vincenzo L Ferrante MS Pellerito V Nardi P Circulating levels of ferritin, RDW, PTLs as predictive biomarkers of postoperative atrial fibrillation risk after cardiac surgery in extracorporeal circulation. Int J Mol Sci. (2022) 23(23):14800. 10.3390/ijms232314800 36499124 PMC9741292

[13] Liu Z Khuong JN Caruana CB Jackson SM Campbell R Ramson DM The prognostic value of elevated perioperative neutrophil-lymphocyte ratio in predicting postoperative atrial fibrillation after cardiac surgery: a systematic review and meta-analysis. Heart, Lung Circ. (2020) 29:1015–24. 10.1016/j.hlc.2019.11.021 32089488

[14] Dey S Kashav R Kohli JK Magoon R Walian A Grover V. Systemic immune-inflammation index predicts poor outcome after elective off-pump CABG: a retrospective, single-center study. J Cardiothorac Vasc Anesth. (2021) 35:2397–404. 10.1053/j.jvca.2020.09.092 33046365

[15] Hu B Yang XR Xu Y Sun YF Sun C Guo W Systemic immune-inflammation index predicts prognosis of patients after curative resection for hepatocellular carcinoma. Clin Cancer Res. (2014) 20:6212–22. 10.1158/1078-0432.CCR-14-0442 25271081

[16] Qi Q Geng Y Sun M Wang P Chen Z. Clinical implications of systemic inflammatory response markers as independent prognostic factors for advanced pancreatic cancer. Pancreatology. (2015) 15:145–50. 10.1016/j.pan.2014.12.004 25641673

[17] Akboga MK Inanc IH Sabanoglu C Akdi A Yakut I Yuksekkaya B Systemic immune-inflammation index and C-reactive protein/albumin ratio could predict acute stent thrombosis and high SYNTAX score in acute coronary syndrome. Angiology. (2023) 74:693–701. 10.1177/00033197221125779 36069742

[18] Zheng P-G Chen P Wang L-J Zhang N. The association of the systemic immune-inflammation index and stent thrombosis in myocardial infarction patients after coronary stent implantation—a retrospectively study. J Thorac Dis. (2023) 15:1726. 10.21037/jtd-23-363 37197550 PMC10183534

[19] Luo Y Zhang J Liu T Yin Z Jin Y Han J The systemic-immune-inflammation index predicts the recurrence of atrial fibrillation after cryomaze concomitant with mitral valve surgery. BMC Cardiovasc Disord. (2022) 22:1–6. 10.1186/s12872-021-02434-3 35152878 PMC8842953

[20] Ata Y Abanoz M. Predictive roles of right coronary artery disease severity and systemic immune inflammation index in predicting atrial fibrillation after coronary bypass operations in patients with right coronary artery disease. Heart Surg Forum. (2021) 24(6):E977–82. 10.1532/hsf.4279 34962463

[21] Selcuk M Cinar T Saylik F Dogan S Selcuk I Orhan AL. Predictive value of systemic immune inflammation index for postoperative atrial fibrillation in patients undergoing isolated coronary artery bypass grafting. Medeniyet Med J. (2021) 36:318. 10.4274/MMJ.galenos.2021.37998 PMC8694166 34939398

[22] Hinoue T Yatabe T Nishida O. Prediction of postoperative atrial fibrillation with the systemic immune-inflammation index in patients undergoing cardiac surgery using cardiopulmonary bypass: a retrospective, single-center study. J Artif Organs. (2023) 26:112–8. 10.1007/s10047-022-01338-z 35579768

[23] Aviles RJ Martin DO Apperson-Hansen C Houghtaling PL Rautaharju P Kronmal RA Inflammation as a risk factor for atrial fibrillation. Circulation. (2003) 108:3006–10. 10.1161/01.CIR.0000103131.70301.4F 14623805

[24] Ömür SE Zorlu Ç Yılmaz M. Comparison of the relationship between inflammatory markers and atrial fibrillation burden. Anatol J Cardiol. (2023) 27:486–93. 10.14744/AnatolJCardiol.2023.2927 37288859 PMC10406144

[25] Zhou X Dudley SC Jr. Evidence for inflammation as a driver of atrial fibrillation. Front Cardiovasc Med. (2020) 7:62. 10.3389/fcvm.2020.00062 32411723 PMC7201086

[26] Warltier DC Laffey JG Boylan JF Cheng DC. The systemic inflammatory response to cardiac surgery: implications for the anesthesiologist. J Am Soc Anesth. (2002) 97:215–52. 10.1097/00000542-200207000-00030 12131125

[27] Asimakopoulos G. Systemic inflammation and cardiac surgery: an update. Perfusion. (2001) 16:353–60. 10.1177/026765910101600505 11565890

[28] Yang B Mustafa RA Bossuyt PM Brozek J Hultcrantz M Leeflang MMG GRADE guidance: 31. Assessing the certainty across a body of evidence for comparative test accuracy. J Clin Epidemiol. (2021) 136:146–56. 10.1016/j.jclinepi.2021.04.001 33864930

[29] Topal D Korkmaz UTK Velioglu Y Yuksel A Donmez I Uçaroğlu ER Systemic immune-inflammation index as a novel predictor of atrial fibrillation after off-pump coronary artery bypass grafting. Revista da Associação Médica Brasileira. (2022) 68:1240–6. 10.1590/1806-9282.20220295 36228255 PMC9575030

[30] Uğuz B Topal D Badem S Kahraman N Uğuz İ. Systemic immune-inflammation index: a novel predictor for risk of postoperative atrial fibrillation in patients undergoing isolated coronary artery bypass grafting. Heart Surg Forum. (2022) 25(5):E665–E73. 10.1532/hsf.4861 36317911

[31] Yilmaz Y Kelesoglu S Elcik D Ozmen R Kalay N. Predictive values of systemic immune-inflammation index in new-onset atrial fibrillation following coronary artery bypass grafting. Brazilian J Cardiovasc Surg. (2022) 38:96–103. 10.21470/1678-9741-2021-0278 PMC10010712 35657307

[32] Filardo G Damiano RJ Jr. Ailawadi G Thourani VH Pollock BD Sass DM Epidemiology of new-onset atrial fibrillation following coronary artery bypass graft surgery. Heart (British Cardiac Society). (2018) 104:985–92. 10.1136/heartjnl-2017-312150 29326112

[33] Phan K Ha HS Phan S Medi C Thomas SP Yan TD. New-onset atrial fibrillation following coronary bypass surgery predicts long-term mortality: a systematic review and meta-analysis. Eur J Cardiothorac Surg. (2015) 48:817–24. 10.1093/ejcts/ezu551 25602053

[34] Eikelboom R Sanjanwala R Le ML Yamashita MH Arora RC. Postoperative atrial fibrillation after cardiac surgery: a systematic review and meta-analysis. Ann Thorac Surg. (2021) 111:544–54. 10.1016/j.athoracsur.2020.05.104 32687821

[35] Thorén E Hellgren L Jidéus L Ståhle E. Prediction of postoperative atrial fibrillation in a large coronary artery bypass grafting cohort. Interact Cardiovasc Thorac Surg. (2012) 14:588–93. 10.1093/icvts/ivr162 22314010 PMC3329319

[36] Lowres N Mulcahy G Jin K Gallagher R Neubeck L Freedman B. Incidence of postoperative atrial fibrillation recurrence in patients discharged in sinus rhythm after cardiac surgery: a systematic review and meta-analysis. Interact Cardiovasc Thorac Surg. (2018) 26:504–11. 10.1093/icvts/ivx348 29161419

[37] Lee SH Kang DR Uhm JS Shim J Sung JH Kim JY New-onset atrial fibrillation predicts long-term newly developed atrial fibrillation after coronary artery bypass graft. Am Heart J. (2014) 167:593–600.e1. 10.1016/j.ahj.2013.12.010 24655710

[38] Rezaei Y Peighambari MM Naghshbandi S Samiei N Ghavidel AA Dehghani MR Postoperative atrial fibrillation following cardiac surgery: from pathogenesis to potential therapies. Am J Cardiovasc Drugs. (2020) 20:19–49. 10.1007/s40256-019-00365-1 31502217

[39] Qureshi M Ahmed A Massie V Marshall E Harky A. Determinants of atrial fibrillation after cardiac surgery. Rev Cardiovasc Med. (2021) 22:329–41. 10.31083/j.rcm2202040 34258901

[40] Mahoney EM Thompson TD Veledar E Williams J Weintraub WS. Cost-effectiveness of targeting patients undergoing cardiac surgery for therapy with intravenous amiodarone to prevent atrial fibrillation. J Am Coll Cardiol. (2002) 40:737–45. 10.1016/S0735-1097(02)02003-X 12204505

[41] Mariscalco G Biancari F Zanobini M Cottini M Piffaretti G Saccocci M Bedside tool for predicting the risk of postoperative atrial fibrillation after cardiac surgery: the POAF score. J Am Heart Assoc. (2014) 3:e000752. 10.1161/JAHA.113.000752 24663335 PMC4187480

[42] Larmann J Theilmeier G. Inflammatory response to cardiac surgery: cardiopulmonary bypass versus non-cardiopulmonary bypass surgery. Best Pract Res Clin Anaesth. (2004) 18:425–38. 10.1016/j.bpa.2003.12.004 15212337

[43] Yamashita K Hu N Ranjan R Selzman CH Dosdall DJ. Clinical risk factors for postoperative atrial fibrillation among patients after cardiac surgery. Thorac Cardiovasc Surg. (2019) 67:107–16. 10.1055/s-0038-1667065 30071562 PMC6359984

[44] Navani RV Baradi A Colin Huang KL Jin D Jiao Y Nguyen JK Preoperative platelet-to-lymphocyte ratio is not associated with postoperative atrial fibrillation. Ann Thorac Surg. (2020) 110:1265–70. 10.1016/j.athoracsur.2020.02.008 32165178

[45] Olesen OJ Vinding NE Østergaard L Butt JH Gislason GH Torp-Pedersen C C-reactive protein after coronary artery bypass graft surgery and its relationship with postoperative atrial fibrillation. Europace. (2020) 22:1182–8. 10.1093/europace/euaa088 32623472

